# Prevalence of Carotid Atherosclerosis in Adult Populations in Europe and North America (USA, Canada): A Systematic Review of Population-Based Studies (2015–2025)

**DOI:** 10.3390/diagnostics16121826

**Published:** 2026-06-12

**Authors:** Maciej Chlabicz, Michał Chlabicz, Wojciech Łaguna, Piotr Myrcha, Jerzy Głowiński

**Affiliations:** 1 Department of Vascular Surgery and Transplantation, Medical University of Bialystok, ul. M. Skłodowskiej-Curie 24A, 15-276 Bialystok, Poland; 2 Population Research Centre, Medical University of Bialystok, 15-269 Bialystok, Poland; 3 1st Chair and Department of General and Vascular Surgery, Faculty of Medicine, Medical University of Warsaw, 02-091 Warsaw, Poland

**Keywords:** carotid atherosclerosis, carotid plaque, population, prevalence, meta-analysis

## Abstract

**Backgrounds:** Carotid atherosclerotic plaques (CAPs) are a reliable marker of systemic atherosclerosis and a predictor of cardiovascular events. Despite advances in prevention, the prevalence of CAPs in high-income regions remains uncertain due to heterogeneity in imaging definitions, study designs, and populations. We strived to provide an updated meta-analysis of population-based studies conducted in Europe and North America between 2015 and 2025, estimating the prevalence of CAPs in general populations. **Methods:** Following the PRISMA 2020 guidelines, PubMed and Web of Science were searched for original studies. Eligible studies reported CAPs prevalence in adult general populations using ultrasonography, computed tomography angiography, and magnetic resonance imaging. Pooled prevalence was calculated using a random-effects meta-analysis of proportions, and heterogeneity was assessed using I^2^ and τ^2^ statistics. Subgroup and meta-regression analyses explored associations with age and comorbidities. **Results:** A total of 80 studies comprising 177,196 participants were included. The pooled prevalence of CAPs was 39.8% (95% CI 32.6–47.5%) under a random-effects model with substantial heterogeneity (I^2^ = 99.6%). The prevalence of CAPs increased with age, exceeding 59% among individuals aged over 70 years. High-risk populations, particularly those with T2DM, exhibited a prevalence exceeding 50%. **Conclusions:** CAPs are present in approximately 40% of adults in Europe and North America, with prevalence strongly driven by age and comorbidities. Despite therapeutic advances, the prevalence of CAPs has not declined, reflecting the growing impact of population aging and comorbidities. Standardized imaging definitions, longitudinal outcome linkage, and pragmatic prevention strategies are needed to translate CAPs detection into reduced cardiovascular events.

## 1. Introduction

Cardiovascular diseases (CVDs) remain the leading cause of morbidity and mortality worldwide, accounting for over 17 million deaths each year [1,2]. One of the key mechanisms leading to cardiovascular events, such as myocardial infarction or stroke, is the development of atherosclerosis. Atherosclerosis is a chronic, lipid-mediated inflammatory disorder affecting medium- and large-caliber arteries, and it is the fundamental pathological substrate of most atherosclerotic cardiovascular diseases (ASCVDs), including coronary artery disease (CAD), ischemic stroke, and peripheral arterial disease (PAD). The process begins with endothelial injury, deposition and modification of low-density lipoprotein in the subendothelial space, followed by monocyte infiltration, foam cell formation, and dysregulated innate and adaptive immune responses. As lesions progress, they are characterized by enlargement of the necrotic core, thinning of the fibrous cap, and development of microcalcifications. This ultimately gives rise to unstable plaques that are predisposed to rupture and thrombosis [3,4]. Early detection and monitoring of its subclinical forms are priorities in the primary and secondary prevention of CVDs and subsequent complications [5]. 

Despite advances in therapy, atherosclerosis remains the leading cause of death in high-income countries. CVDs continue to be the foremost cause of mortality in the United States of America and Europe [6]. In a 2024 report by the American Heart Association (AHA), the age-adjusted cardiovascular mortality rate was 224.3 per 100,000 population, with men experiencing substantially higher rates than women (273.9 vs. 183.1 deaths per 100,000, respectively) [7,8]. In Europe, CVDs account for over 3 million deaths each year across the European Society of Cardiology (ESC) member countries, equivalent to approximately 37.4% of all deaths. The proportion of all deaths due to CVDs is higher in middle-income ESC countries than in high-income ones [9].

Advances in non-invasive imaging techniques allow for early detection of subclinical atherosclerosis and enable more precise CVD risk stratification in asymptomatic individuals. Special emphasis is placed on evaluating carotid atherosclerotic plaques (CAPs), which are easily accessible for non-invasive examination and are recognized as a reliable indicator of systemic atherosclerosis [10]. The presence of CAPs independently predicts CV events and enhances risk stratification for CAD [11,12]. Techniques such as ultrasonographic (USG) carotid plaque assessment, Doppler mode ultrasound, and coronary artery calcium (CAC) scoring can highlight differences between conventional risk estimates and the actual burden of arterial disease, thereby helping to inform decisions about when and how intensively to initiate drug therapy in patients with borderline or intermediate risk profiles. Advanced modalities such as computed tomography angiography (CTA) and magnetic resonance imaging (MRI) are increasingly applied to characterize CAPs. A persistent challenge, however, is the lack of uniform plaque definitions, ranging from focal intima–media thickening >1.5 mm to percent-based thresholds of luminal narrowing. To harmonize practice, the Mannheim Consensus recommends defining plaque as a focal structure encroaching ≥0.5 mm into the lumen, ≥50% thicker than the adjacent IMT, or ≥1.5 mm in wall thickness [13].

Multimorbidity is associated with increased mortality and greater health care needs [14]. Carotid atherosclerosis is especially prevalent in high-risk groups such as individuals with type 2 diabetes mellitus (T2DM) and type 1 diabetes mellitus (T1DM) or a history of CVDs [15]. Both the AHA and the ESC emphasize that individuals with these conditions exhibit a high prevalence of well-established causal risk factors of cardiovascular events compared with the general population, reflecting the cumulative burden of hypertension, dyslipidemia, obesity, and systemic inflammation [16].

Despite abundant data, heterogeneity remains considerable, driven by differences in inclusion criteria, imaging modality, segment coverage, and plaque definition. Estimates vary from 30–40% in community samples to ≥70–80% in older or high-risk groups, emphasizing the need for standardized definitions and imaging protocols to strengthen comparability and meta-analytic precision.

The purpose of this meta-analysis is to quantify the prevalence of CAPs in the general population and to delineate differences in prevalence between apparently healthy cohorts and individuals with type 2 diabetes mellitus (T2DM), type 1 diabetes mellitus (T1DM) and human immunodeficiency virus (HIV) infection. Our findings may provide valuable insights for clinicians and researchers, offering a more precise understanding of the distribution of carotid atherosclerosis.

## 2. Materials and Methods

This systematic literature review followed the guidelines outlined in the Preferred Reporting Items for Systematic Reviews and Meta-Analyses (PRISMA) 2020 Statement Table S1 [17]. Between 2 March 2025 and 8 March 2025, the PubMed and Web of Science databases was queried for original articles written in English and published between 2015 and 2025 using the following search terms: “carotid atherosclerosis” [Title/abstract] AND “prevalence” [Title/abstract] AND “population” [Title/abstract].

The meta-analysis included original scientific papers:

- Conducted in European and North American countries;
- Published in English between 2015 and 2025;
- Containing data on the prevalence of CAPs in adult human population.

The meta-analysis excluded studies:

- Conducted on populations outside Europe and North America;
- Conducted on children or animals;
- In which the prevalence of CAPs was not present or was not measured.

**Figure 1:**
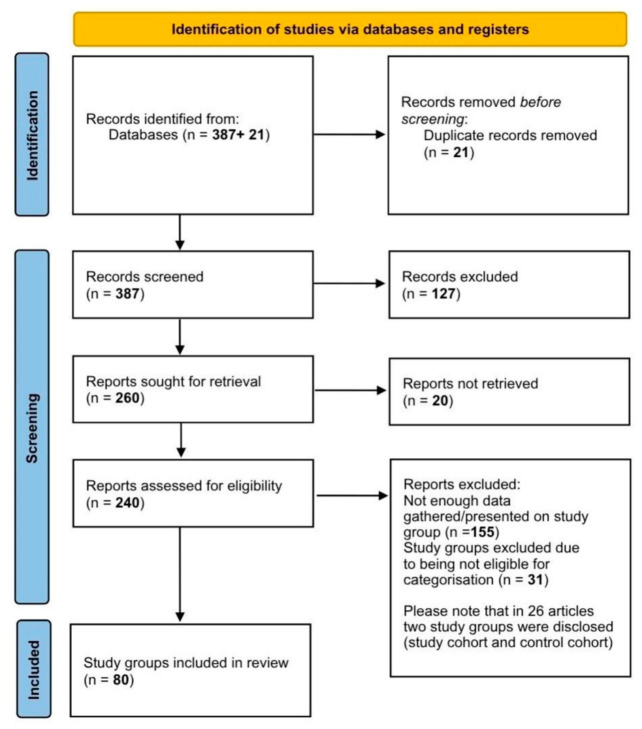
PRISMA 2020 flow diagram of study selection.

As a result, 408 publications were obtained, of which 21 were duplicates. After the initial elimination of 127 papers whose titles and abstracts indicated analyses in areas other than those of interest to the authors, 240 were retrieved, although the full versions of 20 articles were not available. After assessment for eligibility, a total of 80 original articles evaluating the prevalence of CAPs in the adult population of Europe and North America, based on population-based studies conducted between 2015 and 2025, were analyzed. The screening process are shown in Figure 1. Data extraction was performed using a standardized protocol. For each eligible study, the following variables were systematically collected: first author and year of publication; country of study population; population characteristics; sample size of the study group and, where applicable, the control group; mean and/or median age of participants; imaging modality employed (B-mode ultrasonography, Doppler ultrasonography, CTA, MRI, or panoramic radiography); definition of CAPs applied (e.g., Mannheim consensus or study-specific criteria); and prevalence of CAPs, expressed as the percentage of individuals with detectable lesions. All data were summarized in Table 1 and Table 2.

Prevalence of CAPs was pooled using meta-analysis of proportions with logit transformation. Both common-effect and random-effects models were fitted, with the latter (restricted maximum likelihood estimator, Hartung–Knapp adjustment) considered primary. Between-study heterogeneity was quantified using I^2^, τ^2^ and prediction intervals were calculated to reflect the expected range of prevalence in future studies. Subgroup analyses were conducted according to population type (general, apparently healthy, T2DM, T1DM, HIV, other chronic conditions), and age-stratified analyses were performed using predefined age bands (<50, 50–59, 60–69, ≥70 years). The effect of age as a continuous predictor was further examined in random-effects meta-regression.

All analyses were performed in Rv4.5.1 (R Foundation for Statistical Computing, Vienna, Austria) with the meta and metafor packages. Forest and bubble plots were generated to display study-level and pooled estimates.

## 3. Results

A total of 46 population-based study groups comprising 177,196 participants from the general population were included in the quantitative synthesis. The range of median ages for the cohorts was between 38 and 72 years. The pooled prevalence of CAPs was 39.8% (95% CI 32.6–47.5%) under a random-effects model (Hartung–Knapp). Between-study heterogeneity was high (I^2^ = 99.7%, τ^2^ = 0.987, *p* < 0.001), indicating substantial variation in prevalence estimates across studies.

**Figure 2:**
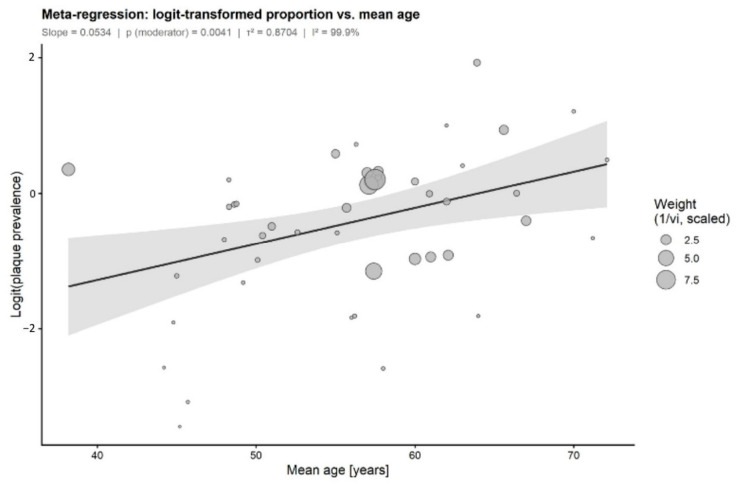
Meta-regression of logit-transformed carotid atherosclerotic plaque (CAP) prevalence by mean/median age across general population-based cohorts.

**Figure 3:**
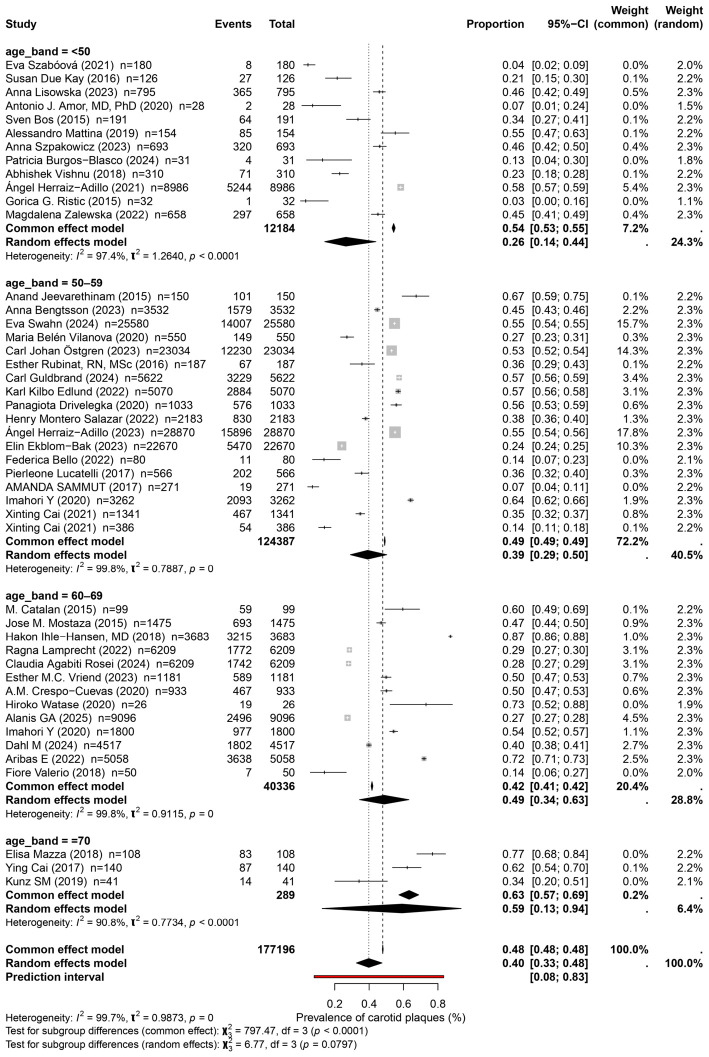
Forest plot of carotid atherosclerotic plaque (CAP) prevalence stratified by age categories across population-based cohorts [18,19,20,21,22,23,24,25,26,27,28,29,30,31,32,33,34,36,37,38,39,40,41,42,43,44,45,46,47,48,49,50,51,52,53,54,55,56,57,58,59,60,85].

Age emerged as a strong determinant of plaque prevalence. In subgroup analyses, the prevalence of CAPs increased progressively across age bands, from 26% in younger adults (<50 years) (95% CI 14–44%), to 39% in middle-aged adults (50–59 years) (95% CI 29–50%), 49% in older middle-aged adults (60–69 years) (95% CI 34–63%), and 59% in older adults (≥70 years) (95% CI 13–94%). In the common-effect model, these differences were significant (*p* < 0.0001); however, they were no longer significant in the random-effects model (*p* = 0.0797), which was used to report the final results. Nevertheless, a meta-regression of mean/median age demonstrated a clear positive association between age and the probability of CAP detection, with each additional decade of age corresponding to a marked increase in pooled prevalence, as shown in Figure 2 and Figure 3.

Meta-regression analyzes identified mean age and comorbidity type as the main moderators of heterogeneity. Each 10-year increase in mean age was associated with a marked rise in the prevalence of carotid plaques (*p* < 0.001). Patients with T2DM exhibited significantly higher prevalence compared to general populations (*p* < 0.01). Neither publication year nor imaging modality showed a significant influence on pooled estimates. The multivariable model combining these moderators reduced between-study variance by approximately one-third, indicating that demographic and clinical characteristics largely explain the observed heterogeneity across studies.

**Figure 4:**
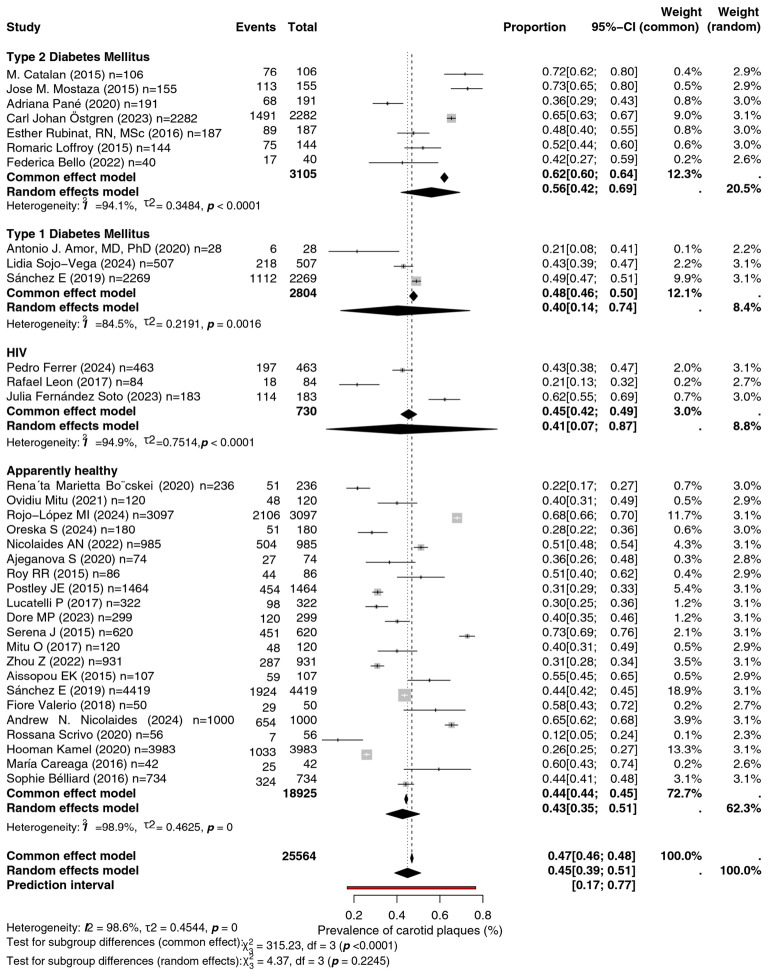
Forest plot of carotid atherosclerotic plaque (CAP) prevalence, stratified by comorbidity groups across population-based cohorts [18,29,61,62,63,64,65,66,67,68,69,70,71,72,73,74,75,76,77,78,79,80,81,82,83,84,86].

In 34 study subgroups analyzed, stratified by underlying clinical condition, the prevalence of CAPs differed significantly among groups. Among patients with T2DM, the pooled prevalence was 56% (95% CI 42–69%). Individuals living with HIV also demonstrated a high but markedly variable prevalence of 41%, with a substantial 95% CI of 7–87%. Overall, these findings indicate that patients with T2DM consistently exhibit the highest prevalence of CAPs, as shown in Figure 4. 

## 4. Limitations

This review is subject to several limitations. First, there is notable methodological heterogeneity across studies. While most investigations relied on B-mode ultrasound and Mannheim consensus criteria, others employed CT or MRI as surrogate methods. Differences in imaging modality, plaque definition, vascular segment coverage, and scoring approaches introduce variability that hampers direct comparison and reduces the precision of pooled estimates. Standardization of definitions and protocols will be essential to improve comparability in future research.

Furthermore, most included studies were cross-sectional, which inherently limits causal inference. While such studies are appropriate for estimating prevalence and identifying associations, they do not allow determination of temporal relationships or causality between risk factors and carotid atherosclerotic plaque development.

**Figure 5:**
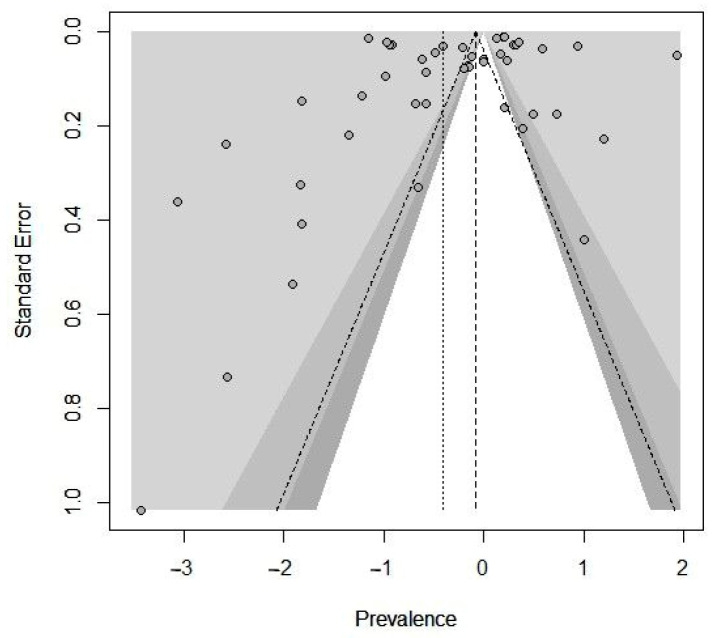
Conventional standard error funnel plot.

**Figure 6:**
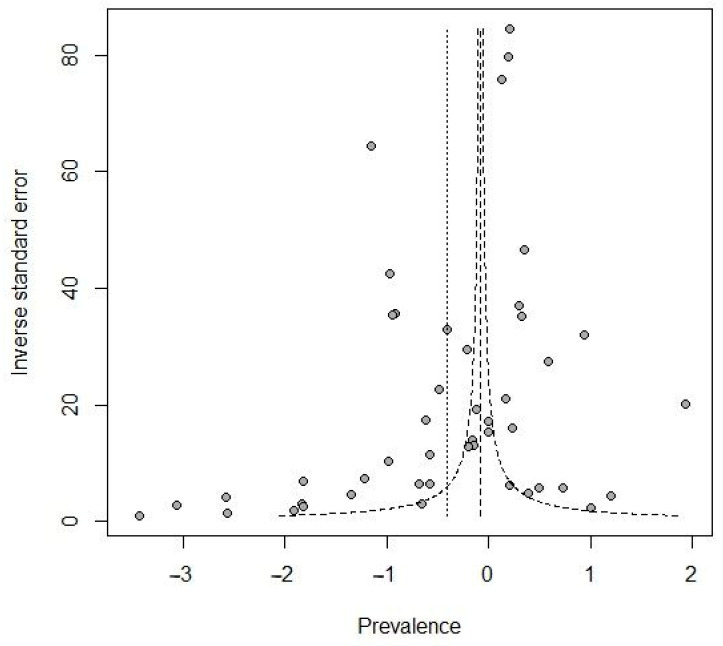
Inverse standard error funnel plot.

To better assess potential publication bias, we generated funnel plots and performed Egger’s regression test, as shown in Figure 5 and Figure 6. Visual inspection of both conventional and inverse standard error funnel plots demonstrated a degree of asymmetry, primarily driven by smaller studies with greater variability and more extreme prevalence estimates, whereas larger and more precise studies remained relatively clustered around the pooled effect size. These findings may suggest small-study effects and potential publication bias; however, they should be interpreted cautiously given the substantial heterogeneity across studies, including differences in imaging modalities, study design, plaque definitions, and population characteristics. In prevalence meta-analyses, funnel plot asymmetry may also reflect genuine clinical and methodological variability rather than selective publication alone. Importantly, despite the visually observed asymmetry, Egger’s regression test did not demonstrate statistically significant publication bias (*p*-value = 0.4968).

In addition, differences in healthcare infrastructure and screening strategies across countries may have affected the reported prevalence of carotid atherosclerotic plaques. Variations in access to imaging modalities, preventive cardiovascular care, referral patterns, and population-based screening practices could partially explain the observed heterogeneity between studies.

Another limitation is the small number of studies including people living with HIV, which resulted in a wide confidence interval (7–87%). This reflects substantial between-study heterogeneity and indicates that the pooled estimate should be interpreted with caution. Therefore, this summary prevalence may not be directly applicable to future populations or different clinical settings. In this context, site-specific estimates should be taken into account when designing screening strategies or planning healthcare delivery for people living with HIV.

## 5. Discussion

Our study revealed that the pooled prevalence of CAPs was 39.8% (95% CI 32.6–47.5%), increasing sharply with age and reaching 59% among individuals aged over 70 years. High-risk populations, particularly those with T2DM, consistently exhibited a higher prevalence, exceeding 50%.

To further explore potential sources of heterogeneity, we performed study-level me-ta-regression analyses, including mean age and geographical region (Southern Europe, Central Europe, Nordic, Central East Europe, and the US) for general population cohorts. Region alone was not a statistically significant moderator of carotid plaque prevalence and explained only a small proportion of between-study heterogeneity. However, in the multivariable model including both mean age and region, the overall model was statistically significant, and mean age remained independently associated with higher plaque prevalence. Specifically, each additional year of mean age was associated with 6.7% higher odds of carotid plaque detection. Nevertheless, substantial residual heterogeneity persisted (residual I^2^ = 99.84%, QE *p* < 0.0001), indicating that other unmeasured clinical, methodological, imaging-related, and healthcare-system factors likely contributed to variability in reported prevalence estimates.

The largest global meta-analysis [87] reported an overall adult prevalence of 21.1% (95% CI 13.2–31.5%) across 59 population-based studies, with higher rates in men and in individuals with CV risk factors. The studies included individuals aged 30–79 years in 2020 from across the world. Differences likely reflect regional risk factor patterns, age structures, and heterogeneous imaging definitions. Importantly, despite advances in prevention and treatment, CAPs prevalence has not declined in high-income settings, underlining the persistence of this subclinical burden. Taken together, global and regional evidence emphasizes the scale of the problem and the urgent need for standardized imaging criteria, longitudinal outcome data, and strategies to incorporate CAP assessment into preventive initiatives.

Findings from several very large, contemporary cohorts substantially reinforce the robustness of our pooled estimates. The Swedish CArdioPulmonary bioImage Study (SCAPIS) is particularly noteworthy. Across multiple analyses including between 25,000 and 28,000 participants aged 50–65 years, the prevalence of CAPs consistently clustered around 50–55% [27]. These estimates were replicated across independent sub-studies, underscoring both internal validity and reproducibility [29,33,35]. An additional SCAPIS sub-study demonstrated that low fitness in adolescence predicted higher plaque burden in midlife, highlighting the importance of preventive strategies across the life course [39]. The Malmö Diet and Cancer–Cardiovascular cohort (MDCS-CV) also provides key population-level evidence. With >4000 participants, CAP prevalence varied between 36% and 61%, depending on the operational definitions applied, again confirming that a substantial proportion of middle-aged adults already carry detectable atherosclerotic disease [88,89]. In Norway, the ACE-1950 cohort offered a unique single-year birth cohort perspective: among over 3600 men and women aged 63–65 years, 87% were found to have carotid plaques [25]. This strikingly high prevalence illustrates the near-universal presence of subclinical atherosclerosis in older middle age, even in the context of modern preventive care. From the United Kingdom, an exceptional dataset of 596,469 individuals who underwent vascular screening revealed a prevalence of 1.87% for carotid stenosis ≥ 50%. Although CAPs were not directly assessed, these data remain informative by quantifying the burden of advanced carotid disease in the general population [90]. Similarly, the REFINE-Reykjavik cohort demonstrated a gradient in plaque burden, with minimal, moderate, and severe plaques in 35%, 9%, and 1% of participants, respectively, confirming strong associations with age, sex, smoking, and T2DM [91]. Together, these studies illustrate how both overall prevalence and severity of CAP scale with age and cardiometabolic burden. Age emerged as the strongest determinant of plaque prevalence, with nearly all studies reporting rates exceeding 70% among adults older than 65 years [22,24,25].

Other studies demonstrate that diabetes markedly amplifies CAP prevalence. In Spain, the rates increased progressively from 34% in control participants to 73% in patients with established T2DM [92]. Comparable patterns were seen in new-onset T2DM [18] and in matched comparisons of T2DM versus controls [62]. Even individuals with prediabetes showed significantly elevated rates of subclinical carotid atherosclerosis, highlighting the importance of early metabolic screening [93,94]. Elevated prevalence has also been documented in other high-risk groups, including patients with chronic kidney disease [95] and HIV infection [80]. Together, these findings highlight the cumulative vascular burden of metabolic and inflammatory related exposures.

Importantly, beyond their epidemiological significance, CAP deposits are a robust diagnostic and prognostic marker of systemic atherosclerosis. There is accumulating evidence that the presence of carotid plaque is associated with an increased risk of future cardiovascular events, including myocardial infarction and ischemic stroke [11]. Data from large prospective cohorts, including MESA, further demonstrate that assessing CAPs improves cardiovascular risk prediction, even in individuals with no coronary artery calcium, highlighting its additional value beyond established imaging markers [11]. Contemporary imaging techniques also enable detailed plaque characterization, including burden, morphology, and features of vulnerability, which are closely linked to adverse cardiovascular outcomes [10]. From a diagnostic perspective, carotid ultrasonography remains a widely accessible, non-invasive and cost-effective tool for detecting subclinical atherosclerosis, allowing early identification of high-risk individuals before clinical events occur.

Bridging the gap between research findings and clinical application, these results highlight the translational potential of CAP detection in everyday practice. The high midlife prevalence of CAPs indicates that noninvasive imaging can meaningfully refine CV risk stratification in primary prevention, especially in individuals at intermediate clinical risk. Visualization of plaques may enhance patient engagement, motivating lifestyle modification and adherence to pharmacotherapy. High-yield populations for targeted screening include midlife adults and patients with diabetes or multimorbidity.

## 6. Conclusions

This meta-analysis provides an updated synthesis of population-based studies assessing CAP prevalence in Europe and North America between 2015 and 2025. CAPs are present in approximately 40% of adults, with prevalence strongly driven by age and comorbidities.

## Figures and Tables

**Table 1:** Data obtained from individual publications—general population cohorts.

First Author	Year of Publication	No of Individuals in a Study Group	No of Individuals with Carotid Plaques	Percent of Individuals with Carotid Plaques [%]	Age Mean or Median ^b^	Methodes: USG—0, USG Doppler—1, CT—2, MRI—3	Are Plaques Measured According to Mannheim Consensus: No—0, Yes—1
M. Catalan [18]	2015	99	59	60.0	63	1	1
J. M. Mostaza [19]	2015	1475	693	47.0	62	1	1
A. Jeevarethinam [20]	2015	150	101	67.3	56	0	1
E. Szabóová [21]	2021	180	8	4.4	46	0	1
E. Mazza [22]	2018	108	83	77.0	70	0	1
S. Due Kay [23]	2016	126	27	21.0	49	0	0
A. Bengtsson [24]	2023	3532	1579	44.7	56	0	0
H. Ihle-Hansen [25]	2018	3683	3215	87.3	64	0	1
A. Lisowska [26]	2023	795	365	45.9	49	0	1
E. Swahn [27]	2024	25,580	14,007	54.8	57	0	1
M. Belén Vilanova [28]	2020	550	149	27.1	50	0	1
C. J. Östgren [29]	2023	23,034	12,230	53.1	57	0	1
E. Rubinat [30]	2016	187	67	35.8	55	0	1
A. J. Amor [31]	2020	28	2	7.1	44	0	1
S. Bos [32]	2015	191	64	33.5	48	0	1
C. Guldbrand [33]	2024	5622	3229	57.4	57	0	1
A. Mattina [34]	2019	154	85	55.0	48	0	1
K. Kilbo Edlund [35]	2022	5070	2884	58.0	58	0	0
A. Szpakowicz [36]	2023	693	320	46.2	49	0	1
P. Drivelegka [37]	2020	1033	576	55.8	58	0	1
H. Montero Salazar [38]	2022	2183	830	38.0	51	0	1
Á. Herraiz-Adillo [39]	2023	28,870	15,896	55.1	58	0	1
E. Ekblom-Bak [40]	2023	22,670	5470	24.0	57	0	1
R. Lamprecht [41]	2022	6209	1772	28.5	62	1	1
F. Bello [42]	2022	80	11	13.8	56	0	0
Ying Cai [43]	2017	140	87	62.1	72	3	0
P. Burgos-Blasco [44]	2024	31	4	12.9	45	0	1
P. Lucatelli [45]	2017	566	202	36.0	53	0	1
A. Vishnu [46]	2018	310	71	22.8	45	0	1
C. Agabiti Rosei [47]	2024	6209	1742	28.0	61	0	1
E. M.C. Vriend [48]	2023	1181	589	49.9	61	0	1
Á. Herraiz-Adillo [39]	2021	8986	5244	58.7	38	0	1
A. Sammut [49]	2017	271	19	7.0	58	2	0
G. G. Ristić [50]	2015	32	1	3.1	45	0	1
A.M. Crespo- Cuevas [51]	2020	933	467	50.1	66	0	1
Hiroko Watase [52]	2020	26	19	73.1	62 ^b^	3	1
S. M. Kunz [53]	2019	41	14	34.1	71	0	1
G. A. Alanis [54]	2025	9096	2496	27.4	60	0	1
Imahori Y ^a^ [55]	2020	3262	2093	64.2	55 ^b^	0	1
Imahori Y ^a^ [55]	2020	1800	977	54.3	60 ^b^	0	1
M. Dahl [56]	2024	4517	1802	40.0	67	0	1
E. Aribas [57]	2022	5058	3638	71.9	66	0	1
F. Valerio [58]	2018	50	7	14.0	64	0	1
M. Zalewska [59]	2022	658	297	45.1	48	0	1
Xinting Cai ^a^ [60]	2021	1341	467	34.9	50	0	0
Xinting Cai ^a^ [60]	2021	386	54	14.0	56	3	0

^a^ Double records are, in fact, studies that include two distinct cohorts. ^b^ Age is presented as a median. CT, computed tomography; MRI, magnetic resonance imaging; USG, ultrasonography imaging.

**Table 2:** Data obtained from individual publications—apparently healthy and clinical comorbidities cohorts.

First Author	Year of Publication	No of Individuals in a Study Group	No of Individuals with Carotid Plaques	Percent of Individuals with Carotid Plaques [%]	Age Mean or Median ^b^	Methodes: USG—0, USG Doppler—1	Population: General—0, T2DM—1, CKD—2, HIV—3, other—4, Apparently Healthy—5, T1DM—6	Are Plaques Measured According to Mannheim Consensus: No—0, Yes—1
M. Catalan [18]	2015	106	76	72.0	62	1	1	1
J. M. Mostaza [19]	2015	155	113	72.9	64	1	1	1
A. Pané [61]	2020	191	68	35.6	47	0	1	1
C. J. Östgren [29]	2023	2282	1491	65.3	59	0	1	1
E. Rubinat [30]	2016	187	89	47.6	57	0	1	1
A. J. Amor [31]	2020	28	6	21.4	46	0	6	1
R. Loffroy [62]	2015	144	75	52.1	60	0	1	0
P. Ferrer [63]	2024	463	197	42.5	49	0	3	1
R. Leon [64]	2017	84	18	21.0	42	0	3	1
J. Fernández Soto [65]	2023	183	114	62.0	52	1	3	1
R. M. Bo¨cskei [66]	2020	236	51	21.6	47	0	5	1
F. Bello [42]	2022	40	17	42.5	59	0	1	0
L. Sojo-Vega [67]	2024	507	218	43.0	49	0	6	1
Ovidiu Mitu [68]	2021	120	48	40.0	52	0	5	1
M.I. Rojo-López [69]	2024	3097	2106	68.0	57	1	5	1
S. Oreska [70]	2024	180	51	28.3	57 ^b^	0	5	1
A.N. Nicolaides [71]	2022	985	504	51.2	58	1	5	0
S. Ajeganova [72]	2020	74	27	35.1	51	0	5	0
R.R. Roy [73]	2015	86	44	51.2	58	0	5	1
J.E. Postley [74]	2015	1464	454	31.0	56	0	5	1
P. Lucatelli [45]	2017	322	98	30.4	52	0	5	1
Dore M. P. [75]	2023	299	120	40.1	65	0	5	1
Serena J. [76]	2015	620	451	72.7	72	1	5	1
Mitu Ovidiu [77]	2017	120	48	40.0	52	0	5	1
Zhou Z. [78]	2022	931	287	30.8	54 ^b^	0	5	1
E. K. Aissopou [79]	2015	107	59	55.0	54	0	5	1
E. Sánchez ^a^ [80]	2019	2269	1112	49.0	59 ^b^	0	6	1
E. Sánchez ^a^ [80]	2019	4419	1924	43.5	57 ^b^	0	5	1
F. Valerio [58]	2018	50	29	58.0	68	0	5	1
A. N. Nicolaides [71]	2024	1000	654	65.4	58	1	5	0
R. Scrivo [81]	2020	56	7	12.5	72	1	5	0
Hooman Kamel [82]	2020	3983	1033	25.9	66	0	5	0
M. Careaga [83]	2016	42	25	60.0	48	0	5	0
S. Bélliard [84]	2016	734	324	44.1	49	0	5	0

^a^ Double records are, in fact, studies that include two distinct cohorts. ^b^ Age is presented as a median. CKD, chronic kidney disease; HIV, human immunodeficiency virus; T1DM, type 1 diabetes mellitus; T2DM, type 2 diabetes mellitus; USG, ultrasonography imaging.

## Data Availability

The original contributions presented in this study are included in the article/Supplementary Materials. Further inquiries can be directed to the corresponding author.

## Supplementary Materials

The following supporting information can be downloaded at: https://www.mdpi.com/article/10.3390/diagnostics16121826/s1, Table S1: PRIMSA 2020 Checklist.

## Abbreviations

The following abbreviations are used in this manuscript:

CAPs	Carotid atherosclerotic plaques
ASCVD	Atherosclerotic cardiovascular diseases
CAD	Coronary artery disease
PAD	Peripheral arterial disease
USG	Ultrasonography
CTA	Computed tomography angiography
MRI	Magnetic resonance imaging
T2DM	Type 2 diabetes mellitus
T1DM	Type 1 diabetes mellitus
HIV	Human immunodeficiency virus
